# First trimester screening for trisomy 21 in gestational week 8-10 by ADAM12-S as a maternal serum marker

**DOI:** 10.1186/1477-7827-8-129

**Published:** 2010-10-29

**Authors:** Niels Tørring, Susan Ball, Dave Wright, Gaïané Sarkissian, Marie Guitton, Bruno Darbouret

**Affiliations:** 1 Department of Clinical Biochemistry, Aarhus University Hospital - Skejby, Aarhus, Denmark; 2 Centre for Health and Environmental Statistics, University of Plymouth, Plymouth, UK; 3 Research and Development Department, Cezanne SAS, Parc Scientifique Georges Besse, Nimes, France

## Abstract

**Background:**

A disintegrin and metalloprotease 12 (ADAM12-S) has previously been reported to be significantly reduced in maternal serum from women with fetal aneuploidy early in the first trimester and to significantly improve the quality of risk assessment for fetal trisomy 21 in prenatal screening. The aim of this study was to determine whether ADAM12-S is a useful serum marker for fetal trisomy 21 using the mixture model.

**Method:**

In this case control study ADAM12-S was measured by KRYPTOR ADAM12-S immunoassay in maternal serum from gestational weeks 8 to 11 in 46 samples of fetal trisomy 21 and in 645 controls. Comparison of sensitivity and specificity of first trimester screening for fetal trisomy 21 with or without ADAM12-S included in the risk assessment using the mixture model.

**Results:**

The concentration of ADAM12-S increased from week 8 to 11 and was negatively correlated with maternal weight. Log MoM ADAM12-S was positively correlated with log MoM PAPP-A (r = 0.39, P < 0.001), and with log MoM free beta hCG (r = 0.21, P < 0.001). The median ADAM12-S MoM in cases of fetal trisomy 21 in gestational week 8 was 0.66 increasing to approx. 0.9 MoM in week 9 and 10. The use of ADAM12-S along with biochemical markers from the combined test (PAPP-A, free beta hCG) with or without nuchal translucency measurement did not affect the detection rate or false positive rate of fetal aneuploidy as compared to routine screening using PAPP-A and free β-hCG with or without nuchal translucency.

**Conclusion:**

The data show moderately decreased levels of ADAM12-S in cases of fetal aneuploidy in gestational weeks 8-11. However, including ADAM12-S in the routine risk does not improve the performance of first trimester screening for fetal trisomy 21.

## Background

The soluble form of A disintegrin and metalloprotease 12 (ADAM12-S) with suggested proteolytic activity on Insulin-like growth factor binding protein (IGFBP) - 3 and 5 and the epidermal growth factor ligands EGF, Betacellulin and HB-EGF [1], is synthesized by the placenta syncytiotrophoblasts [2,3] and is present in high concentrations in maternal serum from early first trimester [4-6]. Since ADAM12-S was first reported to be significantly reduced in maternal serum from women with fetal trisomy 21 [4] much attention has been given to investigating the utility of ADAM12-S as a prenatal marker for fetal aneulpoidy in first and second trimester [4-9], fetal pre-eclampsia [10,11] and intra uterine growth restriction [12]. The discriminatory efficiency of ADAM12-S as a marker for fetal aneuploidy in first trimester has been inconclusive. Reported median multiple of median (MoM) for maternal serum ADAM12-S in mothers with fetal trisomy 21 in first trimester have ranged from being substantially lower than those in unaffected pregnancies to showing relatively small reductions. Laigaard *et al.*, first reported a median ADAM12-S MoM value of just 0.140 among 18 cases of trisomy 21, collected largely before 10 weeks gestation [4]. Screening algorithms that included ADAM12-S in addition to both maternal age and first trimester biochemistry and maternal age, biochemistry and NT showed that ADAM12-S was a potentially valuable addition to first trimester screening. Subsequent studies [4,13-15], which have included serum samples collected both early and late in the first trimester, have suggested that ADAM12-S is less promising as an effective marker for fetal trisomy 21. Median ADAM12-S MoM values for trisomy 21 pregnancies in these studies are largely inconsistent with the initial findings of Laigaard *et. al.*, and show a moderate to negligible reduction relative to unaffected MoM values; ranging from 0.61 MoM [16] among samples collected before 10 weeks gestation, to 0.977 MoM [15] between 11 and 13 weeks gestation. Large variation in the reported ADAM12-S MoM values and in screening test results has failed to confirm the utility of ADAM12-S as a prenatal marker, either alone or combined with pregnancy-associated plasma protein-A (PAPP-A) and maternal serum free beta-human chorionic gonadotropin (free beta-hCG) and in conjunction with Nuchal Translucency (NT). Whether the diversity in published results represents differences in preanalytical handling of clinical samples [13] and/or immunoassay performance remains to be investigated.

In order to develop further our understanding of ADAM12-S as a marker for fetal aneuploidy in first trimester and to determine the clinical potential of this marker in routine screening for fetal trisomy 21, we have examined the maternal serum concentration of ADAM12-S using a newly developed immunoassay in 46 samples of maternal serum from women with fetal trisomy 21, and 645 controls in gestational week 8-11.

## Methods

### Serum samples

This was a case-control study using samples that were collected as part of the nationwide prenatal screening program in Denmark. Blood samples were drawn by family general practitioners (GPs) as part of the routine first trimester screening program from gestational week 8+0 to 13+6. Serum was isolated and sent to the Department of Clinical Biochemistry at Aarhus University Hospital - Skejby for analysis. All samples were stored at -80°C and were thawed prior to analysis of ADAM12-S. 46 samples of maternal serum from genetically verified fetal trisomy 21 between gestational week 8+1 and 11+6 were analysed. 42 of these were identified by the routine first trimester risk assessment program based on preclinical risk, PAPP-A, free β-hCG and NT using a risk cut-off of 1 in 300 at the time of analysis. 4 cases of fetal trisomy 21 were not identified by the prenatal screening program and resulted in live-born infants. As a control group 645 samples of maternal serum from mothers from gestational week 8+0 to 11+6 with unaffected spontaneous conceived, singleton pregnancies from non-diabetic mothers with different parity were used. Data collected on maternal characteristics included maternal weight, smoking status and ethnicity; all women were Caucasian, non-smokers. PAPP-A and free β-hCG results determined by the Brahms KRYPTOR assay were available on all samples. Ultrasound examination was performed during gestational weeks 11 + 2 to 13 + 6. Gestational age was determined by measurement of CRL using the formula obtained by Robinson [17], and the gestational age at time of blood sampling was calculated accordingly. Only blood samples with a gestational age between 8+0 and 11+6 were used for the study. The mean maternal ages were 31 years (controls) and 35 years (trisomy 21). The use of samples for the purpose of method development in first trimester screening has been approved by the Danish Central Biomedical Research Ethics Committee, Journal No. 2006-7041-88.

### Assay procedures, antibodies, standards and controls and testing

Serum ADAM12-S was measured using the Brahms KRYPTOR using a newly developed assay for the purpose of routine biochemical screening (Cezanne SAS, Nimes, France). The assay was set up a homogenous sandwich fluoroimmunoassay using time resolved amplified cryptate emission (TRACE) technology [18]. In the assay, purified anti-human ADAM12-S rat monoclonal antibodies of clones 41R and 29R were coupled to AF647 fluorophore (Invitrogen, San Diego, CA) and to europium cryptate TBP-Di-SMP (Cis Bio International SAS, Saclay, France), respectively.

Controls were prepared from a third-trimester serum pool diluted (495 μg/L and 2295 μg/L) in horse serum (Sigma H 1138). We calibrated a third-trimester serum pool against recombinant ADAM12-S, and used the pool to generate a standard curve for determining ADAM12-S concentrations. The KRYPTOR ADAM12-S has an assay range of 240 to 10 267 μg/L and the dilution curves are linear in this concentration range (r^2 ^> 0.99). The mean recovery of concentrated ADAM12-S added to serum samples was 109% (range 102-118%, n = 4). Intra-assay variation was determined to be 5.2%. The average inter-assay variations in 6 runs were from 1.8% to 7.7%.

### Statistics

Multiple regression modelling of log transformed ADAM12-S, PAPP-A and free β-hCG concentrations was used to obtain log MoM values specific to gestational age and maternal weight. Adequacy of fit was assessed using diagnostic plots of MoM values.

Screening performance results are based on patient-specific risks for trisomy 21, calculated from the product of the maternal age-related risk and the likelihood ratio (LR) for the appropriate combination of biochemical markers.

Where NT has been included in the screening algorithm, the patient-specific risks have been calculated by multiplying the maternal age-related risk by the LR for the biochemistry and the LR for NT, as defined in the mixture model [19].

Empirical screening performance results include maternal age standardised detection and false-positive rates (with 95% confidence intervals) for tests incorporating (i) PAPP-A and free beta-hCG, (ii) PAPP-A, free beta-hCG and ADAM12-S, (iii) PAPP-A, free beta-hCG and NT, (iv) PAPP-A, free beta-hCG, NT and ADAM12-S, (v) PAPP-A and ADAM12-S and (vi) PAPP-A, ADAM12-S and NT, for risk cut-offs of 1 in 100 and 1 in 250 at the time of screening. The maternal age distribution of England and Wales in the three years from 2000 to 2002 [20] was used as a reference for estimating the standardised rates.

Modelled screening performance results are based on the simulation of 100 000 unaffected and 100 000 trisomy 21 pregnancies with the 2000 to 2002 maternal age distribution of England and Wales, NT distributions according to the mixture model and distributions of log MoM ADAM12-S, log MoM PAPP-A and log MoM free β-hCG derived from the data.

## Results

ADAM12-S was analysed in 46 cases of fetal trisomy 21 and 645 controls between gestational age 8+0 and 11+6.

**Figure 1:**
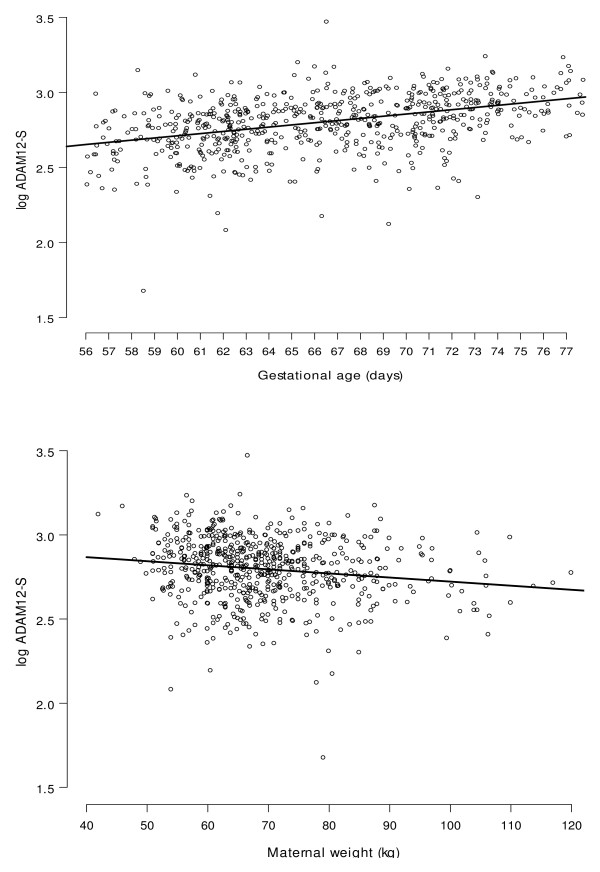
**ADAM12 concentration in serum is dependent on Maternal Weight**. Log ADAM12-S concentration (μg/l) in maternal serum from 645 controls by gestation (top panel) and by weight (bottom panel). The superimposed lines correspond to the fitted relationship from the multiple regression model **log ADAM12-S = 2.9530 + 0.01454 (gestational age (days) - 77) - 0.002421 (maternal weight - 69)**. The median maternal weight (67 kg) and median gestational age (66 days) have been used to obtain the fitted lines on the top and bottom panels respectively.

**Figure 2:**
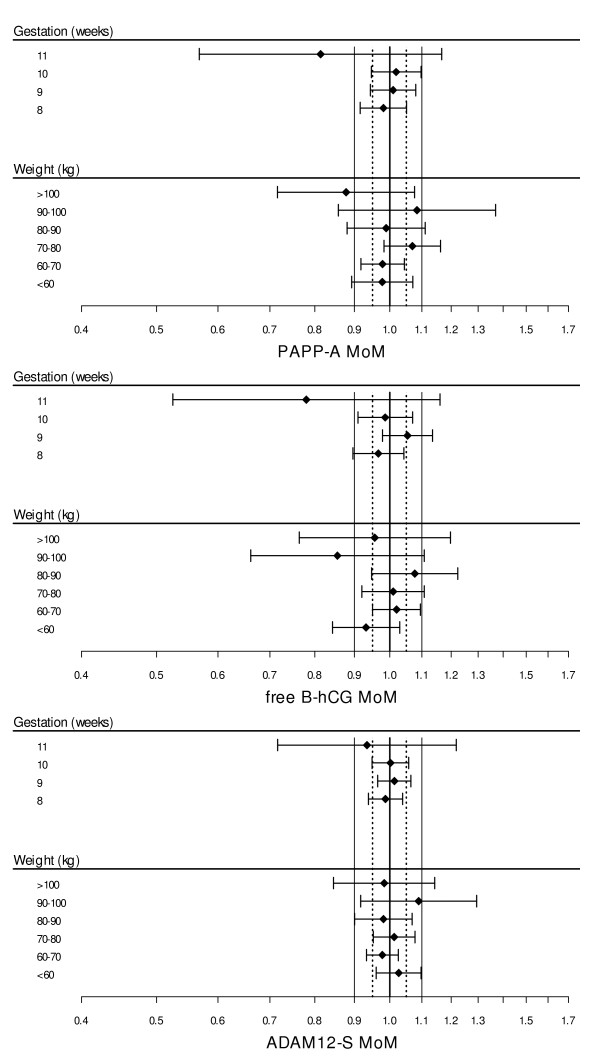
**Biochemical markers in unaffected pregnancies**. Diagnostic plots for ADAM12-S, PAPP-A and free β hCG MoM values in unaffected pregnancies.

In the controls the median concentration (μg/l) of ADAM12-S increased throughout the three weeks. Figure 1 shows the relationship between ADAM12-S and gestational age (top panel) and ADAM12-S and maternal weight (bottom panel). Multiple regression analysis showed significant effects (P < 0.001) on the concentration of ADAM12-S from both gestational age and maternal weight and the fitted relationship from the multiple regression model is superimposed on the plots in Figure 1. The median MoM values for ADAM12-S, PAPP-A and free beta-hCG at 8 to 11 weeks gestation and for maternal weight show an adequate model fit in each case (Figure 2).

The correlations between log MoM ADAM12-S, PAPP-A and free βhCG in unaffected pregnancies (by gestational week) and in trisomy 21 pregnancies are given in Table 1. Notable features of Table 1 are the strong positive correlations between log MoM ADAM12-S and log MoM PAPP-A in the control pregnancies, with minimal variation across gestational age, and the significant positive correlation (r = 0.33, P < 0.03) between log MoM ADAM12-S and log MoM PAPP-A in trisomy 21 pregnancies.

**Table 1:** Correlations between biochemical markers in first trimester screening

	Controls		trisomy 21 cases
	gestational week	correlation	correlation
ADAM12-S and PAPP-A	8	0.40	
	9	0.34	
	10	0.43	
	Overall	0.39	0.33
ADAM12-S and free β-hCG	8	0.30	
	9	0.10	
	10	0.28	
	Overall	0.21	-0.02

Correlations between log MoM ADAM12-S and PAPP-A and log MoM ADAM12-S and free β-hCG in unaffected pregnancies (by gestational week) and in trisomy 21 pregnancies.

There was a general decrease in concentration of ADAM12-S in cases of fetal aneoploidy compared to the controls with similar gestational age at time of blood sampling. The median ADAM12-S MoM of the trisomy 21 cases was 0.66 in gestational week 8 increasing to 0.95 in week 9 and 0.85 in week 10, with standard deviations between 0.11 and 0.16 (Table 2). Log MoM ADAM12-S values in the fetal trisomy 21 cases was described by the linear regression: log MoM ADAM12-S = -0.004227 + 0.007818 × (gestational age (days) - 77).

**Table 2:** ADAM12 Median MoM in Cases (fetal trisomy 21) and Controls

Weeks	Controls median MoM	SD (95% CI)	n	Trisomy 21 median MoM	SD (95% CI)	n
8+0 to 8+6	0.992	0.173 (0.158 - 0.190)	217	0.664	0.158 (0.118 - 0.241)	17
9+0 to 9+6	1.008	0.173 (0.158 - 0.190)	229	0.945	0.143 (0.107 - 0.214)	18
10+0 to 10+6	1.003	0.153 (0.139 - 0.170)	191	0.848	0.111 (0.078 - 0.195)	11
11+0 to 11+6	0.932	0.147(0.100-0-299)	8	-	-	-

Median MoM and SD log MoM ADAM12-S in controls (n = 645) and trisomy 21 cases (n = 46) in gestational week 8 to 11.

SD: standard deviation, CI: confidence interval.

The detection rates and false-positive rates from including and excluding ADAM12-S, for risk cut-offs of 1 in 100 and 1 in 250 at time of screening, are given in Table 3. Table 4 shows detection rates for a fixed false-positive rate of 5%. These have been, standardised to a reference maternal age population (UK 2000 to 2002). The corresponding modelled results using the parameter estimates from the data and assuming the fitted multivariate Guassian distributions are given in Tables 5 and 6.

**Table 3:** Empirical results

	1 in 100		1 in 250	
	FPR (95% CI)	DR (95% CI)	FPR (95% CI)	DR (95% CI)
**(i) maternal age, PAPP-A & free B-hCG**	2.9 (1.5, 4.2)	72.2 (65.7, 78.7)	9.4 (7.0, 11.8)	86.2 (79.0, 93.3)
**(ii) maternal age, PAPP-A, free B-hCG & ADAM12-S**	2.6 (1.3, 3.9)	73.7 (67.7, 79.6)	9.3 (7.0, 11.7)	87.3 (80.4, 94.2)
**(iii) maternal age, PAPP-A, free B-hCG & NT**	0.6 (0.1, 1.2)	71.1 (61.8, 80.5)	2.8 (1.5, 4.1)	83.7 (75.7, 91.8)
**(iv) maternal age, PAPP-A, free B-hCG, NT & ADAM12-S**	0.7 (0.1, 1.3)	71.1 (61.8, 80.5)	2.6 (1.4, 3.8)	87.7 (80.4, 94.9)
**(v) maternal age, PAPP-A & ADAM12-S**	2.5 (1.4, 3.7)	56.8 (50.1, 63.5)	9.2 (6.9, 11.4)	73.7 (63.6, 83.8)
**(vi) maternal age, PAPPA, ADAM12-S & NT**	1.4 (0.6, 2.3)	75.1 (67.2, 83.1)	3.5 (2.2, 4.9)	81.4 (75.3, 87.6)

Results for a screening test including maternal age, PAPP-A and ADAM12-S, with and without NT. These results are empirical values, standardised to a reference maternal age population (UK 2000 to 2002). Detection rates and false-positive rates for fetal trisomy 21 at fixed risk cut-offs of 1 in 100 and 1 in 250 are given. Figures in brackets are 95% confidence intervals. Substantial decrease in DR when first trimester free B-hCG in (i) is replaced with ADAM12-S to give (v), with a negligible decrease in FPR.

Substituting free B-hCG in the combined test (iii) for ADAM12-S to give (vi), gives a slightly higher DR and FPR at a risk cut-off of 1 in 100, and a slightly lower DR and higher FPR at a risk cut-off of 1 in 250.

**Table 4:** Detection rate for a fixed false-positive rate: Empirical results

	DR (95% CI)
**(i) maternal age, PAPP-A & free B-hCG**	76.5 (69.4, 83.7)
**(ii) maternal age, PAPP-A, free B-hCG & ADAM12-S**	73.7 (67.7, 79.6)
**(iii) maternal age, PAPP-A, free B-hCG & NT**	95.8 (94.6, 97.1)
**(iv) maternal age, PAPP-A, free B-hCG, NT & ADAM12-S**	92.9 (88.0, 97.8)
**(v) maternal age, PAPP-A & ADAM12-S**	61.6 (52.1, 71.1)
**(vi) maternal age, PAPP-A, ADAM12-S & NT**	83.2 (77.7, 88.8)

Results for first trimester screening test with and without ADAM12-S. These results are empirical values, standardised to a reference maternal age population (UK 2000 to 2002).

Detection rates for fetal trisomy 21 at a fixed false-positive rate of 5% are given. Figures in brackets are 95% confidence intervals. Reduction in DR when ADAM12-S is added to first trimester biochemistry and when ADAM12-S is added to the combined test. Substantial reduction in DR when free B-hCG in (i) is replaced with ADAM12-S to give (v) and when free B-hCG in (iii) is replaced with ADAM12-S to give (vi).

**Table 5:** Modeled results

	1 in 100		1 in 250	
	FPR	DR	FPR	DR
**(i) maternal age, PAPP-A & free B-hCG**	4.6	77.7	9.8	87.3
**(ii) maternal age, PAPP-A, free B-hCG & ADAM12-S**	4.6	77.6	9.8	87.2
**(iii) maternal age, PAPP-A, free B-hCG & NT**	2.5	87.0	5.1	91.9
**(iv) maternal age, PAPP-A, free B-hCG, NT & ADAM12-S**	2.5	86.9	5.2	92.0
**(v) maternal age, PAPP-A & ADAM12-S**	5.4	72.3	11.9	84.3
**(vi) maternal age, PAPP-A, ADAM12-S & NT**	2.8	84.1	6.0	90.0

Results for a screening test including maternal age, PAPP-A and ADAM12-S, with and without NT. Modeled detection rates and false-positive rates for fetal trisomy 21 at fixed risk cut-offs of 1 in 100 and 1 in 250.

Increase in FPR and decrease in DR when free B-hCG in (i) is replaced with ADAM12-S to give (v) and when free B-hCG in the combined test (iii) is replaced with ADAM12-S to give (vi).

**Table 6:** Detection rate for a fixed false-positive rate: Modelled results

	DR
**(i) maternal age, PAPP-A & free B-hCG**	78.5
**(ii) maternal age, PAPP-A, free B-hCG & ADAM12-S**	78.6
**(iii) maternal age, PAPP-A, free B-hCG & NT**	91.6
**(iv) maternal age, PAPP-A, free B-hCG, NT & ADAM12-S**	91.7
**(v) maternal age, PAPP-A & ADAM12-S**	71.0
**(vi) maternal age, PAPP-A, ADAM12-S & NT**	88.9

Results for first trimester screening test with and without ADAM12-S. Modelled detection rates for fetal trisomy 21 at a fixed false-positive rate of 5%.

Virtually no change in DR when ADAM12-S is added to first trimester biochemistry and when ADAM12-S is added to the combined test.

Substantial reduction in DR when free B-hCG in (i) is replaced with ADAM12-S to give (v) and also reduction in DR when free B-hCG in (iii) is replaced with ADAM12-S to give (vi).

Including ADAM12-S in the risk calculations affects neither the detection rate nor the false positive rate at fixed risk cut offs of 1 in 100 and 1 in 250, and with a fixed false positive rate both with and without inclusion of NT in the risk calculations. Exchanging free βhCG with ADAM12-S also did not affect the performance of the risk assessment.

## Discussion

The results of this study show that ADAM12-S has little value as a marker of fetal trisomy 21 early in first trimester from gestational age 8+0 to 11+0. We used the KRYPTOR ADAM12-S assay which has not previously been validated for this purpose. The present assay is based on the TRACE^® ^technology, employing two antibodies that recognized two different epitopes in disintegrin domain of the ADAM12-S molecule. The results show a high reproducibility with SD log MoM values around 0.15-0.17 for the controls and 0.11 to 0.16 for the cases. This performance is in agreement with previous data [21].

The results show an increase in the concentration of ADAM12-S over the three weeks, which has previously been reported using other assays [5,6]. However the absolute concentrations of ADAM12-S determined by the KRYPTOR ADAM12-S assay are somewhat higher as compared to previous data [9,16,22,23]. None of the assays which have been used for quantitative measurement of ADAM12-S are traceable to international standard preparations of ADAM12-S, and therefore not directly comparable. However, using the multiple of median (MoM) to express the relative value of ADAM12-S in serum samples from women with fetal aneuploidy allows us to compare the results of the present study with others. In the group of fetal trisomy 21 we found a median MoM in gestational week 8 of 0.66 increasing to around 0.9 in week 9-10. This is comparable to previous studies [5,6,13]. Together, these studies include more than 340 cases of fetal trisomy 21 early in the first trimester and strengthen the impression that ADAM12-S is only moderately decreased early in the first trimester, with the discriminatory value of this marker weakening with increasing gestational age in the first trimester.

Our results support previous studies that have described a positive correlation between ADAM12-S and PAPP-A early in the first trimester [5,24,25]. Although ADAM12-S MoM is decreased in the first trimester, the strong positive correlation between ADAM12-S and PAPP-A in maternal serum negatively affects the efficacy of ADAM12-S as a marker in first trimester screening. Addition of ADAM12-S to the risk calculation using the mixture model does not increase the detection rate or lower the screen positive rate of fetal trisomy 21, since the discriminatory value is already encountered in the PAPP-A likelihood ratio. The present results describing the lack of efficacy of ADAM12-S on the detection rate and false positive rate of screening for fetal trisomy 21 in the first trimester emphasises that ADAM12-S cannot generally improve the screening for fetal trisomy 21 early in the first trimester.

## Conclusion

According to the literature ADAM12-S has previously been reported to significantly improve the detection rate and false positive rate of first trimester screening for fetal trisomy 21 [4,13]. However the present results and a number of other large studies have failed to confirm these data, and indicate that ADAM12-S is only moderately decreased in cases of fetal trisomy, and does not improve the performance of first trimester screening for fetal trisomy 21.

## Competing interests

NT, SB and DW report neither financial nor non-financial competing of interest. GS, MG and BD are employed by Cezanne SAS which has developed the assay for measurement of ADAM12. The authors alone are responsible for the content and writing of the paper, and the content and the conclusions in the manuscript are not affected by the financial interest.

## Authors' contributions

NT coordinated the design of the study, the collection of results and writing the manuscript. SB and DW performed the statistical analysis, and helped to draft the manuscript. MG carried out the immunoassay and statistical data analysis. GS and BD participated in the design of the study and helped to draft the manuscript. All authors read and approved the final manuscript.
